# Clinical and translational medicine in Europe – horizon 2020 and beyond

**DOI:** 10.1186/1479-5876-10-S2-A5

**Published:** 2012-10-17

**Authors:** Roland Andersson

**Affiliations:** 1 Lund University, Vice Dean (Innovation, Translational Research), Medical Faculty, Lund, Sweden

## Background

Health providers and thereby academy and industry is challenged by gradually increasing demands from not at least an ageing population, including increases in cancer, cardiovascular and neurological diseases, diabetes, and complex disease patterns with multimorbidity. At the same time, economical restraints will not allow the society to increase resources spent on health care.

## Methods

The effects of the ageing population, changes in life style (overweight, obesity, poor dietary habits, lack of physical activity) needs to be dealt with. Demands from patients are to improve quality of care and adding life to years instead of just adding to your life. To provide European citizens with lifelong health and wellbeing, visions for horizon 2020 (and beyond) has been performed (Copenhagen Research Forum). The global evolution in biomedicine will provide access to new technologies that will require implementation. At the same time, as health care is a factor for investment including industry, education and training is also a possibility to create innovation and work in research and improved health.

## Results

Preventive measures will future on have large impact on the health care situation and both clinical and translational research, including e.g. lifestyle, food intake, environmental factors. The willingness from the society to spend more money on health care will be limited. The ageing population will face advanced home care using remote health monitoring, smart phones with medical apps etc, rendering communication with health care providers. Bioinformatics, advanced diagnostics, novel combinations of therapies and improved knowledge on the individual biological phenotypes (genomics, proteomics, metabolomic profiles) will drive diagnosis and treatment towards personalized medicine. The previously long established “one-size fits all” concept will be replaced by individualized and tailored management, i.e. a true paradigm shift. Biobanks will be of substantial help, providing e.g. novel biomarkers for diagnosis and therapy.

Novel technologies bridging the fields of medicine and technology/chemistry will provide us with e.g. nanomedicine, both for imaging and treatment, as well as artificial, bioartificial and tissue engineered organs.

## Conclusions

The economical restraints future on will force us to translational and clinical research towards personalized medicine (diagnostics and treatment), thereby steering efficient therapy e.g. with companion diagnostics and increasing cost effectiveness in health care. This paradigm shift will demand academy and industry to provide completely novel tools and thereby possibilities for innovation and potential commercialization within life science in close collaboration between health care, academy, and industry, focusing on the patient’s need.

